# Supplementary material for: Whole-Genome Sequencing for Resistance Prediction and Transmission Analysis of Mycobacterium tuberculosis Complex Strains from Namibia
Source: Microbiol Spectr. 2022 Sep 27;10(5):e01586-22. doi: 10.1128/spectrum.01586-22 (PMC9603870; doi:10.1128/spectrum.01586-22)
Supplement: Supplemental file 3 — Legends of Tables S1 and S2. Download spectrum.01586-22-s0003.pdf, PDF file, 0.07 MB [file spectrum.01586-22-s0003.pdf]

**Supplemental Material**

**Table S1.** Mutation catalogue on which the resistance prediction is based on.

**Table S2.** Data table with all information about the cohort, as well as information of the MTBC strains including lineage definition, called mutations in resistance genes and clustering.
